# Expanding the phenotype of *CRYAA* nucleotide variants to a complex presentation of anterior segment dysgenesis

**DOI:** 10.1186/s13023-020-01484-8

**Published:** 2020-08-13

**Authors:** Andrey V. Marakhonov, Anna A. Voskresenskaya, Maria Jose Ballesta, Fedor A. Konovalov, Tatyana A. Vasilyeva, Fiona Blanco-Kelly, Nadezhda A. Pozdeyeva, Vitaly V. Kadyshev, Vanesa López-González, Encarna Guillen, Carmen Ayuso, Rena A. Zinchenko, Marta Corton

**Affiliations:** 1 Research Centre for Medical Genetics, Moskvorechie Str., 1, Moscow, Russian Federation; 2 Cheboksary Branch of the S. Fyodorov Eye Microsurgery Federal State Institution, Cheboksary, Russian Federation; 3 Medical Genetics Department, University Hospital Virgen de la Arrixaca, Murcia, Spain; 4 Center for Biomedical Network Research on Rare Diseases (CIBERER), ISCIII - Instituto de Salud Carlos III, Madrid, Spain; 5 Independent Clinical Bioinformatics Laboratory, Moscow, Russian Federation; 6 Department of Genetics & Genomics, Instituto de Investigación Sanitaria-Fundación Jiménez Díaz University Hospital, Universidad Autónoma de Madrid (IIS-FJD, UAM), Av. Reyes Católicos n° 2, 28040 Madrid, Spain

**Keywords:** *CRYAA*, Microphthalmia, Microcornea, Congenital aphakia, NGS, Anterior segment dysgenesis, Aniridia

## Abstract

**Background:**

Mutations in *CRYAA*, which encodes the α-crystallin protein, are associated with a spectrum of congenital cataract–microcornea syndromes.

**Results:**

In this study, we performed clinical examination and subsequent genetic analysis in two unrelated sporadic cases of different geographical origins presenting with a complex phenotype of ocular malformation. Both cases manifested bilateral microphthalmia and severe anterior segment dysgenesis, primarily characterized by congenital aphakia, microcornea, and iris hypoplasia/aniridia. NGS-based analysis revealed two novel single nucleotide variants occurring de novo and affecting the translation termination codon of the *CRYAA* gene, c.520T > C and c.521A > C. Both variants are predicted to elongate the C-terminal protein domain by one-third of the original length.

**Conclusions:**

Our report not only expands the mutational spectrum of *CRYAA* but also identifies the genetic cause of the unusual ocular phenotype described in this report.

## Background

Embryonic lens development is a critical step during eye organogenesis, especially of the anterior segment [1]. Congenital aphakia is a rare developmental disorder characterized by the absence of the lens. Primary aphakia arises in the first 4 weeks of embryogenesis from a lack of lens induction caused by genetic defects, teratogenic effects or rubella virus infection [2–4]. Secondary congenital aphakia occurs as a result of spontaneous resorption or intrauterine rejection of the developing lens [5]. Congenital aphakia is usually reported in association with other severe ocular conditions, such as the sclerocornea–microphthalmia–aphakia complex caused by *FOXE3* mutations [6].

Lens crystallins (α-, β-, and γ-) are the major components of lens fiber cells and are required for maintaining the clarity and refractive properties of the lens [7, 8]. In addition to the function of crystallins as molecular chaperones regulating correct protein folding [9], several crystallins are expressed early during lens organogenesis and fiber cell differentiation, acting as apoptotic regulators [7]. Molecular defects in several crystallin genes lead to congenital cataracts. Missense mutations in the *CRYAA* gene, which encodes αA-crystallin, lead to autosomal dominant congenital cataract with or without microcornea (OMIM #604219) [10, 11], as well as to rare and severe presentations of cataract, microphthalmia, and iris coloboma [12].

**Fig. 1:**
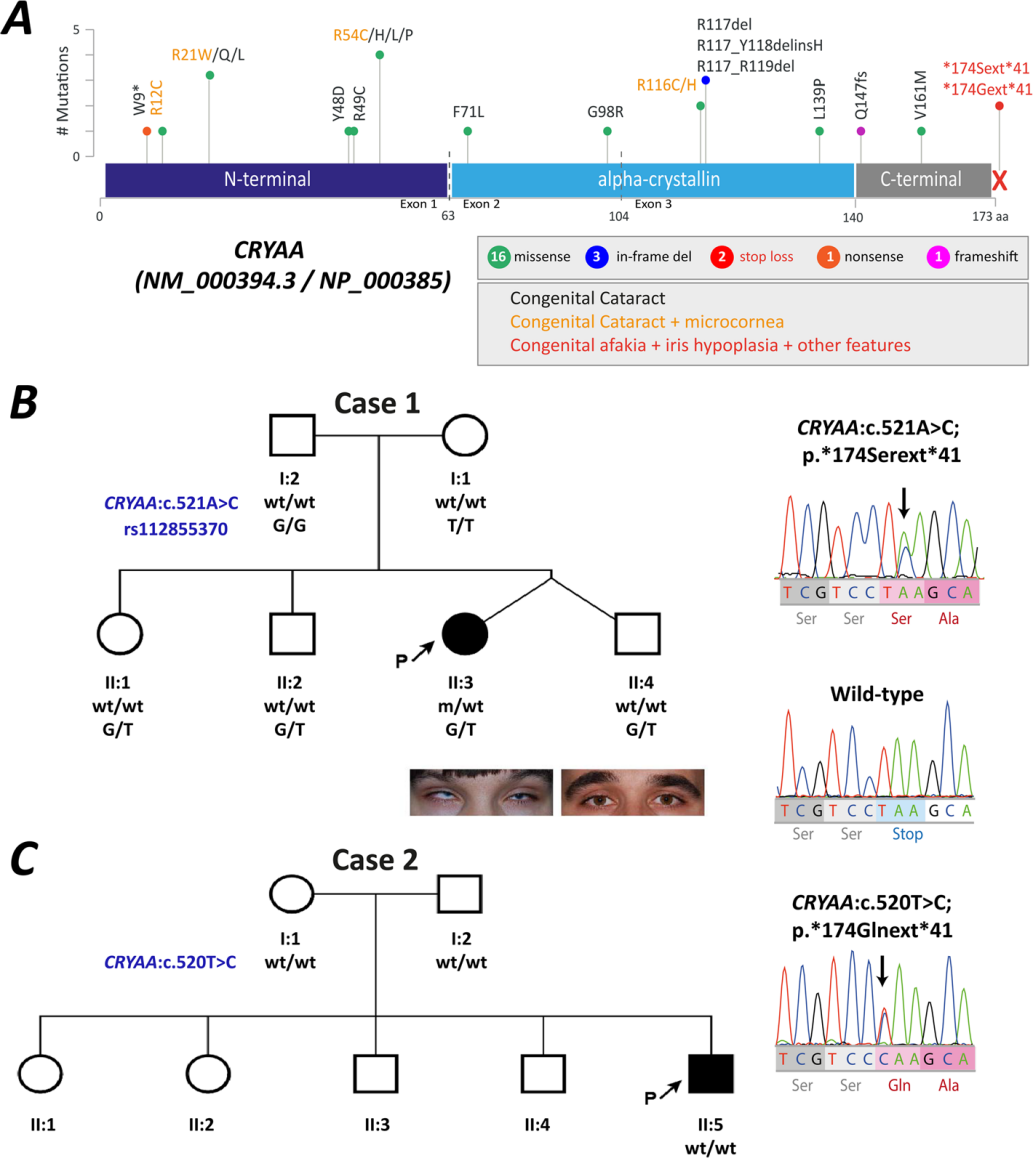
**a**. Schematic representation of the CRYAA mutational spectrum. The upper and lower sections show the position of the exons and the protein structure with three CRYAA protein domains, respectively. The two novel stop-loss variants associated with a complex phenotype of congenital aphakia are represented in red. The pathogenic variants previously described in the literature for congenital cataracts or congenital cataract–microcornea syndrome are represented in black and orange, respectively. The number and the type of variants located in each amino acid residue are also indicated. Pedigrees and familial segregation of the two families (**b** and **c**) carrying de novo run-on mutations in *CRYAA*. Arrows indicate the probands (P); wt/wt represents wild-type individuals; mut/wt indicates heterozygous individuals

To date, 15 missense mutations, 3 small in-frame deletions, and one frame-shifting variant affecting *CRYAA* are registered in publicly available databases as being associated with autosomal dominant congenital cataract with or without microcornea (OMIM #604219) [10, 11]. An additional nonsense variant p.(Trp9*) demonstrates an autosomal recessive mode of inheritance; thus, it has been described only in the homozygous state [13]. Missense variants are thought to produce a relatively mild phenotype of congenital cataract related to a dominant negative mechanism of *CRYAA*-associated cataractogenesis [14]. Most of these changes are at hotspots and alter highly conserved arginine residues located on the N-terminal and crystallin domains (Fig. 1a). Some of these mutations, such as p.(Arg12Cys), p.(Arg12Leu), p.(Arg21Trp) and p.(Arg116Cys), have also been associated with a more complex phenotype of congenital cataracts with microphthalmia and microcornea and in some cases in association with other developmental defects of the anterior segment, primarily with iris coloboma [12, 15–18]. Additionally, microcornea and congenital vascularized corneal opacity have also been associated with p.(Arg116His) [19]. Thus, αA-crystallin seems to play a role in anterior segment development that has not been identified to date.

In this study, we identified two novel read-through *CRYAA* variants as causes of a novel complex phenotype, primarily characterized by congenital aphakia, microphthalmia, iris hypoplasia and posterior anomalies. This gene was not previously associated with complex panocular maldevelopment; therefore, we expanded not only the mutational spectrum of *CRYAA* but also the associated phenotypes.

## Results

In this study, we describe two unrelated sporadic cases from different geographical origins (Russia and Spain), presenting a notably rare form of microphthalmia and congenital aphakia. Both patients share a similar panocular phenotype of bilateral microphthalmia and severe anterior segment dysgenesis, primarily characterized by congenital aphakia, microcornea, and iris hypoplasia. The primary ophthalmological findings of both patients are summarized in Table 1.

**Table 1:** Ophthalmological features of the patients carrying CRYAA mutations

***Case***	1	2	Yamamoto, 1988 (index case)
***Country of origin***	Russia	Spain	Japan
***Age (y) at last ophthalmic revision***	27	8 (only LE)^a^	49
***Genetic change***	c.521A > C; p.(*174Serext*41)	c.520T > C; p.(*174Glnext*41)	Unknown
***Segregation***	de novo	de novo	Apparently dominant inheritance
***BCVA (RE/LE)***	LP / 20/400	NA / 20/400	LP / NLP
***Refraction***	+ 8.5	+ 15	NA
***Microphthalmia***	+ (B)	+ (B)	No
***Anterior-posterior axis (mm) (RE/LE)***	18.67 / 19.06	NA / 13	23.4 / 23.7
***Lens***	Congenital aphakia (B)	Congenital aphakia (B)	Membranous structure (reabsorbed cataract)
***Iris***	Hypoplasia (B)	Hypoplasia (B)	Totally absent iris (LE), rudimentary iris (RE)
***Cornea***	Microcornea (B), increased thickness (B)	Microcornea, congenital corneal edema, and cornea opacity (B)	Microcornea with corneal opacity (B)
***Cornea diameter (mm)***	6.0 × 6.5 (B)	NA	10.0 × 10.5 (B)
***Central corneal thickness (μm)***	790 / 820	NA	NA
***Posterior segment***	Optic nerve and foveal hypoplasia (B)	Intravitreous hemorrhages (B), retinal detachments and atrophy (RE), and glaucomatous cup (LE)	Foveal hypoplasia, glaucomatous cup, and dot hemorrhages (RE)
***Nystagmus***	+ (B)	+ (B)	+ (B)
***Glaucoma***	+ (B)	+ (LE)	+ (B)
***IOP (mm Hg) (RE/LE)***	33 / 33	4 / 8^a^	70 / 70
***Other features***	Strabismus	Congenital leukocoria, anterior PFV and hypotalamia (B)	Altered ERG
***Qx interventions***	None	Membranectomy, pupiloplasty and anterior vitrectomy (B), orbital prosthesis (RE), and Ahmed valve implant (LE)	Laser membranectomy and trabeculectomy (RE)

*Notes:* ^a^*RE not assessable (prosthetic eye).*

*B* bilateral*, BCVA* best corrected visual acuity*, ERG* electroretinogram, *IOP* intraocular pressure*, LE* left eye*, LP* light perception*, NA* not available*, NLP* no light perception*, PFV* persistent fetal vasculature*, Qx* Surgical interventions*, RE* right eye, *y* years

**Fig. 2:**
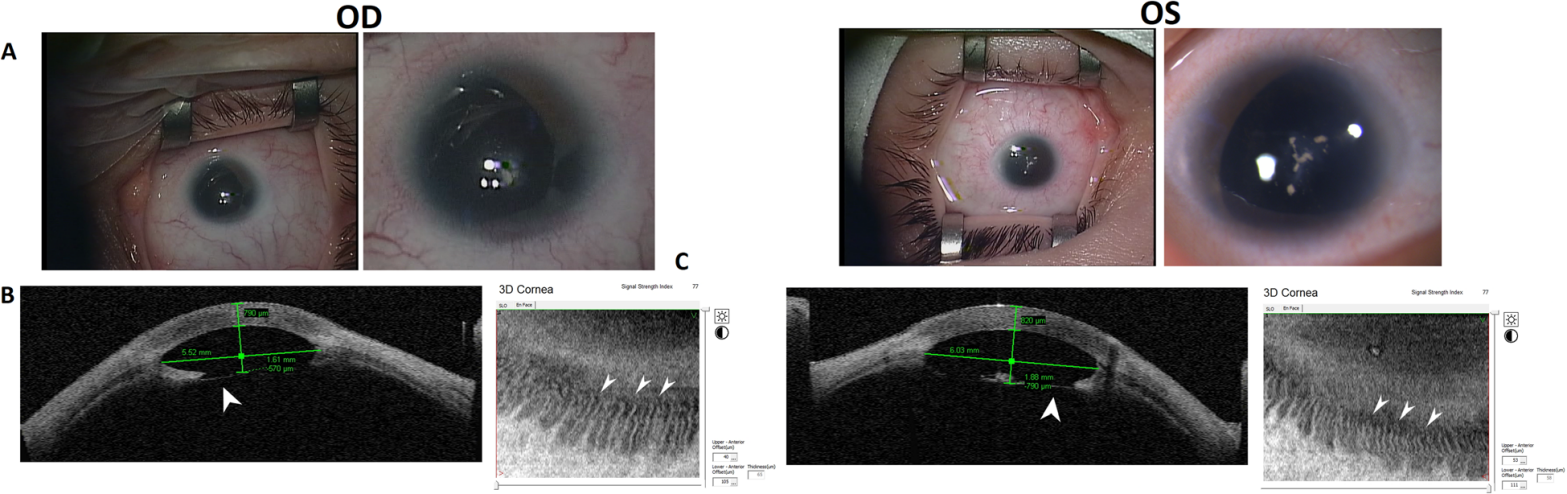
Examination data for Case 1. **a** Images demonstrating a significant decrease in corneal dimensions, rudimentary remnants of the iris on the temporal side, and local opacity in the plane of the absent lens. **b** AS-OCT images (Visante OCT, Carl Zeiss, Germany) showing significant reduction in the size of the anterior chamber of the eye, increase in corneal thickness, lens capsule remnants, and absence of a formed lens (indicated by arrows). **с** AS-OCT images of the inferior limbus in EnFace mode (RTVue XR Avanti, Optovue, USA): hyperreflective parallel lines are seen that correspond to the limbal palisades of Vogt (indicated by arrows)

***Case 1*** is a 27-year-old Caucasian female with bilateral microphthalmia, microcornea, partial aniridia, congenital aphakia, nystagmus, strabismus, secondary glaucoma, and fovea and optic disc hypoplasia. The patient was born as a result of dizygotic twin pregnancy in a family without ophthalmological pathology (Fig. 1b). Data for the presence of intrauterine infection were not available. She was born at full term following a normal pregnancy. Ophthalmic examination revealed a decrease in the corneal dimensions, an increase in corneal thickness and lack of limbal stem cell deficiency. Anterior segment optical coherence tomography (AS-OCT) confirmed the presence of limbal palisades of Vogt in the inferior half of the limbus (Fig. 2), a marked reduction in the anterior chamber size, rudimentary temporal iris remnants and absence of a formed lens. However, the lens capsule was visualized in both eyes, with a 1.5-mm diameter opaque lens substance being retained in the central optical zone and a Y-shaped suture in the left eye (LE) (Fig. 2). Fundus examination showed pale and small optic nerve, lack of foveal depression, and the intersection of large retinal vessels of the macular area. To date, no eye surgery has been performed. No extraocular anomalies were observed. No reliable data on the presence of the lens in this patient in childhood are available, but current ophthalmic data (discovery of the capsular bag and the remnants of the fetal lens nucleus) suggested that aphakia might be secondary, since the initial steps of lens development had occurred, and both lenses were reabsorbed. Ophthalmological examination of her brother (dizygotic twin) revealed only slight pigmentary dispersion on the anterior lens capsule with preserved visual acuity (20/20) in both eyes (Fig. 2). Her parents and other healthy siblings did not show ophthalmic or systemic pathology after clinical examination.

***Case 2*** is a 10-year-old Caucasian boy with bilateral microphthalmia, persistent fetal vasculature (PFV), microcornea, iris hypoplasia, congenital primary aphakia, and secondary glaucoma. He is the fifth child of unrelated healthy parents with no family history of congenital abnormalities or ocular diseases. He was born at full term after an uneventful pregnancy with a birth weight of 3.82 kg. Ophthalmological examination at birth showed bilateral congenital leukocoria, corneal edema, severe hypotalamia (grade 2), lack of formed lens, severe anterolental PFV, dense pupillary membrane, iris hypoplasia, and typical iridohyaloid vessels. Biomicroscopy and ultrasound exams showed microcornea with severe opacity and retinal detachment of the right eye (RE) and dyscoria pupil and corneal edema of the left eye (LE). Fundus examination of LE revealed a pale and excavated optic disk; the RE was not assessable. Bilateral membranectomy, pupiloplasty, and anterior vitrectomy were performed at the age of 1 month without intraocular lens implantation. After this intervention, the patient developed several complications, including intraocular hemorrhages and retinal detachments in RE that were derived to a pre-phthisis state. Consequently, an external orbital prosthesis was implanted to correct his facial asymmetry at 9 months of age. LE had several self-releasing episodes of intravitreous hemorrhages and developed secondary glaucoma that was not controlled with hypotensive treatments. At 7 years of age, a second surgery was performed for implantation of an Ahmed glaucoma valve in the temporal superior sector of LE and for corneal scraping of band keratopathy. The last LE exam at 8 years of age showed horizontal pendular nystagmus and best-corrected visual acuity of 20/400 (corrected with + 15 DS) that remained stationary for the last 4 years. He had normal psychomotor and cognitive development.

In both cases, next-generation sequencing (NGS) identified two likely pathogenic heterozygous substitutions in *CRYAA* affecting different nucleotides of the termination codon*.* In case 1, whole exome sequencing (WES) found only 6 candidate variants in 6 genes after variant filtering (Suppl Table 1). However, according to the gene function and the associated phenotypes, only the *CRYAA* variant NM_000394.4:c.521A > C, which changes the translation termination codon to serine p.(*174Serext*41), was the most likely cause (Fig. 1b). No other likely pathogenic variants in eye developmental genes were identified. In case 2, a custom capture-based NGS panel of 121 genes involved in eye developmental disorders identified the variant NM_000394.4:c.520T > C that leads to a similar stop-loss change p.(*174Glnext*41) (Fig. 1c). No additional likely pathogenic variants were identified.

Both variants are predicted to lead to a C-terminal extension (CTE) of 41 residues affecting the well-conserved polar domain of αA-crystallin (Fig. 1a). These variants were classified as likely pathogenic variants with the criteria PM2, PM4, PP3, PS2 using the American College of Medical Genetics (ACMG) recommendations (Table 2). First, both variants are novel, given their absence in population databases or in specific healthy controls from the same ethnic backgrounds. Additionally, familial segregation with confirmed paternity and maternity defined that both variants occurred as de novo events (Fig. 1b), also supporting their likely causality for disease presentation. Specifically, haplotype analysis of the variant c.521A > C and a closely linked SNP (rs112855370) enabled us to determine that this pathogenic variant occurred de novo in the paternal allele (Fig. 1b). Several in silico tools predicted that both variants are likely damaging. Splicing effects were discarded by using several in silico tools.

**Table 2:** In silico predictions for the *CRYAA* variants

***Variant***	***Inheritance***	***gnomAD Freq***	***ACMG classification (criteria)***	***GERP***	***PhyloP100***	***CADD***	***DANN***	***FATHMM-MKL***	***Mutation*** ***Taster***	***Eigen***	In silico ***splicing prediction***	***SIFT***	***Align*** ***GV-GD***
c.520T > C; p.(*174Glnext*41)	de novo	None	Likely pathogenic (PM2, PM4, PP3, PS2)	3.7899	2.87	15.34	0.8224	P (0.7147)	B	P (0.6226)	No effect on splicing	NA	NA
c.521A > C; p.(*174Serext*41)	de novo	None	Likely pathogenic (PM2, PM4, PP3, PS2)	3.7899	2.963	14.26	0.6924	P (0.7147)	B	P (0.6226)	No effect on splicing	NA	NA

*Notes: V*ariants were numbered according to RefSeq transcript NM_000394.4 for *CRYAA*.

*B* benign, *NA* not available, *P* pathogenic

## Discussion

To the best of our knowledge, no cases of a combination of congenital aphakia, iris hypoplasia, microphthalmia, and microcornea have been associated with *CRYAA* mutations. In addition to isolated congenital cataracts, defects in several lens crystallins may also lead to presentations of microphthalmia and cataracts, with or without microcornea, e.g., heterozygous missense *CRYAA* mutations [16, 17] and a homozygous *CRYBB2* variant [14, 20]. Other phenotypes of cataract, microcornea, and anterior segment dysgenesis have also been associated with *PAX6* and *MAF* variants [21–24]. However, the most important distinguishing features of the phenotype described in this study are the remarkable lens absence, as well as the existence of panocular anomalies also affecting the posterior eye segment. In the Russian patient, the presence of lens remnants of nuclear matter, Y-shaped sutures, and some transparent peripheral parts of the lens capsule may indicate intrauterine lens underdevelopment along with the preservation of the fetal nucleus, rather than spontaneous lens resorption. Thus, aphakia seemed to be secondary but still congenital. Similar phenotypes of congenital aphakia, microphthalmia, and anterior segment dysgenesis have been only reported for some *FOXE3* and *PAX6* mutations, two transcription factors involved in lens vesicle formation [3, 25, 26]. Mutations in connexin *GJA8* have also been recently associated with lens maldevelopment in patients with congenital aphakia or rudimentary lenses [24]. Three decades ago, Yamamoto et al. described a three-generation Japanese family in which at least 3 individuals manifested an ocular presentation resembling those described in this report [27]. In this family, the phenotypic description (OMIM 106230) was referred to as aniridia, microcornea, and spontaneously reabsorbed cataract. The common features of both the cases presented by us and the familial case by Yamamoto et al. are the panocular lesions of the eye, the absence of signs of somatic diseases and mental retardation in patients. The phenotypic heterogeneity of Japanese cases varies depending on the severity of corneal opacity, the degree of preservation of iris tissue and the angle structures of the anterior chamber of the eye and the lens (Table 2). At the same time, the Japanese authors do not report the presence of microphthalmia and persistent fetal vasculature in their patients. Anamnestic data of several generations of the same family indicate cataract development at an early age, while slit-lamp examination visualizes a sufficiently large volume of preserved opaque cortical lens masses both in the optical center and at the periphery of the lens. The author describes such opacities as a membranous or spontaneously reabsorbed cataract. Although lens maldevelopment is also a shared characteristic, the timing of lens resorption in the Japanese family has not been determined. Therefore, it is not possible to conclude that it was congenital. No genetic data were reported for this family; thus, *CRYAA* mutations should be taken into consideration. In the cases we have described, OCT data and eye photographs enable us to visualize accurately the transparency of the peripheral parts of the lens without preserving the cortical masses, which makes it possible to understand early resorption of the lens material, possibly during its intrauterine development, making an aphakia to be secondary.

In this study, we identified for the first time two different *CRYAA* variants affecting the stop codon and likely leading to a similar CTE of 41 amino acid residues, which correspond to approximately one-third of the total protein length. Few variants have been identified in the C-terminus of *CRYAA*. We presume that in our cases, the severity of the damage to the eye structures might be associated with the mutation type affecting the *CRYAA* gene. This C-terminal domain seems to be important not only for protein solubility but also for the chaperone activity of αA-crystallin [9]. Functional studies and protein alignment analysis orthologous to crystallins suggest that an aberrant extension of the polar C-terminal domain of αA-crystallin would have a strong cytotoxic effect affecting all eye structures [28]. Consistent with this hypothesis, CTE mutations in other crystallins associated with congenital cataract usually cause more severe phenotypes than intragenic mutations. Thus, a 26 amino acid residues CTE caused by a read-trough mutation in *CRYBB1* (NM_001887.3:c.757T > C, p.*253Argext*26) resulted in congenital cataract–microcornea syndrome by increasing βB1-crystallin hydrophobicity and promoting aggregate formation [28, 29]. The C-terminal domain plays an important role in the function of αA-crystallin as a survival protein, having a cytoprotective function in several ocular cells [30–32]. This domain was described as essential in preventing Bax-induced apoptosis [31, 32]. Remarkably, Bax- and Bak-deficient mice present PFV [33], which is likely related to apoptotic defects of hyaloid vessels. Strikingly, failure of fetal vasculature regression is one of the characteristic features of the patient carrying the variant c.520T > C. Other possible pathogenic mechanisms underlying CTE mutations might be related to the apoptosis function of αA-crystallin during eye development. Further experiments are needed to test these hypotheses.

## Conclusions

Since *CRYAA* is expressed early in the lens placode prior to exerting a structural role in developing fiber lenses [34], our work provides additional evidence for the involvement of αA-crystallin in lens formation. Furthermore, the results of our study may help to elucidate additional functions of crystallin in cell survival or autophagy regulation in lens epithelium and primary fiber cell differentiation, as well as fetal vasculature regression [35, 36]. In addition, the intact lens orchestrates proper differentiation of different anterior segment structures through molecular signals from the lens epithelium [37]. Therefore, our findings also support the hypothesis that *CRYAA* could not influence lens embryogenesis but may also participate in the maldevelopment of different anterior segment structures, such as the cornea and iris.

## Patients and Methods

### Patient cohort and samples

Case 1 was recruited as part of a Russian study of anterior segment dysgeneses approved by the Institutional Review Board of the Research Centre for Medical Genetics (Moscow, Russia). The affected proband and her healthy relatives underwent detailed ophthalmic examination in the Cheboksary Branch of the S. Fyodorov Eye Microsurgery Federal State Institution (Cheboksary, Russia). AS-OCT (RTVue XR Avanti, Optovue, USA) using 3D Cornea (En Face mode) was performed as described previously [38].

Case 2 was recruited as part of a Spanish study of congenital ocular anomalies approved by the Ethics Committee of the Fundación Jiménez Díaz University Hospital, Madrid, Spain. The affected proband and his healthy progenitors were clinically studied at the University Hospital Virgen de la Arrixaca, Murcia, Spain.

Written informed consent was obtained from all individuals involved in the study (or their legal guardians) prior to their participation. Genomic DNA was obtained using standard procedures from peripheral blood samples.

### Clinical genetic testing

Following routine genetic testing, both patients had previously been screened for the most prevalent aniridia-related gene, *PAX6* (Case 1), or microphthalmia-related genes, such as *OTX2* and *SOX2* (Case 2), as described previously [39, 40]. In both cases, no pathogenic variants were found.

#### Whole-exome sequencing (case 1)

WES was performed using an Illumina NextSeq 500 instrument with an average on-target coverage of 146× with Nextera Rapid Capture Exome v1.2 reagents (Illumina, San Diego, CA) for library preparation (Genomed Ltd., Moscow, Russia). Bioinformatic analysis was performed using an in-house software pipeline designed to detect both single-nucleotide variants (SNVs) and copy number variations (CNVs) as described [41]. Further filtering was performed by functional consequences and population frequencies (gnomAD AF < 0.5% and < 0.1% for recessive and dominant genes, respectively), as well as clinical relevance according to the Human Phenotype Ontology database [42].

#### Targeted resequencing (case 2)

Targeted resequencing of 121 genes associated with eye developmental diseases was performed as previously reported [43]. Capture libraries for all coding and noncoding exons, including 20 bp of intronic boundaries, were obtained using HaloPlex technology (Agilent Technologies, Santa Clara, CA). The libraries were pooled and sequenced on an Illumina NextSeq 500 instrument with 150-bp paired-end reads. Bioinformatic analysis was performed using standard procedures and custom in-house pipelines for both SNVs and CNVs, as previously described [43]. Variant prioritization was performed considering only novel variants or those with a minor frequent allele < 0.5% and < 0.1% for recessive and dominant genes, respectively, in the Genome Aggregation Database (gnomAD, http://gnomad.broadinstitute.org/) and the Spanish Variant Server (http://csvs.babelomics.org/). Potentially functional SNVs and indels in coding regions were considered. In both cases, pathogenicity prediction analysis of the novel variants was carried out using Combined Annotation Dependent Depletion (CADD, http://cadd.gs.washington.edu/), GERP, PhyloP100, DANN, FATHMM-MKL, Mutation Taster and Eigen from Varsome [44], as well as splicing tools (Human Splicing Finder 3.1, Splicing Sequence Finder, MaxtEntScan, NNSPLICE) on Alamut software (Interactive Biosoftware, France) as well as newer tools based on neural networks (SpliceAI [45] and MMSplice [46]). The identified variants were classified for pathogenicity following the recommendations of the American College of Medical Genetics and Genomics (ACMG) [47].

#### Sanger sequencing

All pathogenic *CRYAA* variants retained after filtering from the WES or targeted resequencing data were confirmed by Sanger sequencing using specifically designed primers for exon 3 (available on request). Parental studies were also performed to determine whether these variants were inherited or appeared de novo. In Case 1, paternity and maternity were confirmed by multiplex analysis of 19 STR loci plus Amelogenin using the COrDIS Plus kit (Gordiz Ltd., Moscow, Russia) on the ABI PRISM® 3130XL Genetic Analyzer (Applied Biosystems Inc., Waltham, MA) according to the manufacturer’s recommendations. In Case 2, paternity was confirmed using quantitative fluorescent PCR analysis of 26 genetic markers on chromosomes 13, 18, 21 and XY (Devyser Compact QF-PCR v3, Devyser AB, Hägersten, Sweden), as previously described [48].

#### PCR-RFLP analysis (case 1)

Polymerase chain reaction–restriction fragment length polymorphism (PCR-RFLP) analysis was used to determine the specific population frequency of the variant c.521A > C (Case 1) on a cohort of 110 healthy control individuals from the same ethnic background as Case 1 (Chuvash, a Turkic ethnic group, which is indigenous in the Chuvash Republic, Russian Federation). The genotyping of the wild-type allele (A) was performed by cutting with endonuclease *Eco*P15I (New England Biolabs, Inc., USA).

Linkage and parental origin of the affected allele for case 1 was performed by PCR-RFLP analysis followed by reamplification of the uncut mutant allele and Sanger sequencing with determination of the rs112855370 genotype, which showed informativity in the family.

## Supplementary information

**Additional file 1.**

## Data Availability

The datasets used and/or analyzed during this study are available from the corresponding author upon reasonable request.
